# Supplementary material for: Anatomical and functional maturation of the mid-gestation human enteric nervous system
Source: Nat Commun. 2023 May 9;14:2680. doi: 10.1038/s41467-023-38293-z (PMC10170115; doi:10.1038/s41467-023-38293-z)
Supplement: Supplementary file 1 — Supplementary Information [file 41467_2023_38293_MOESM1_ESM.pdf]

Supplementary Figures

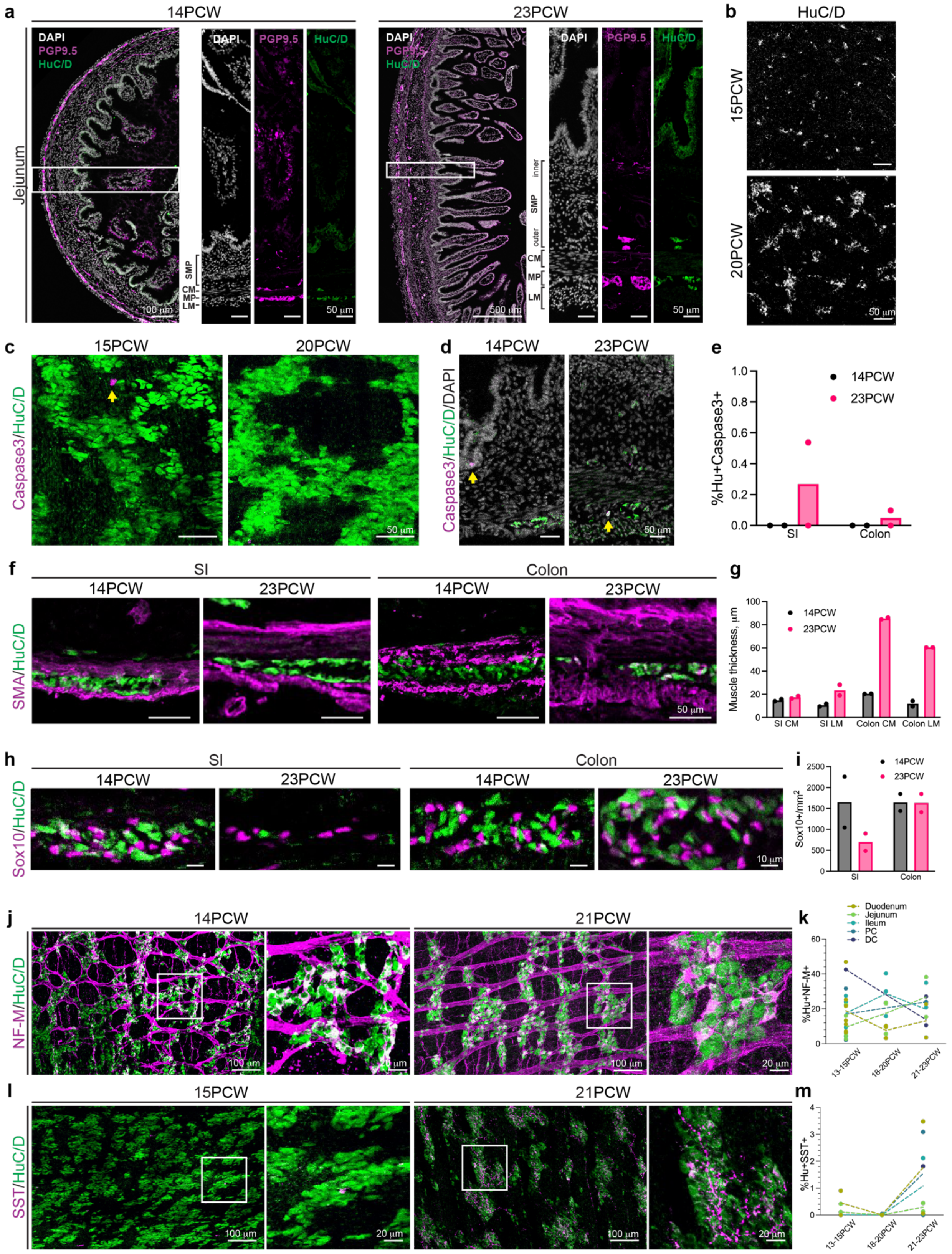

Supplementary Figure 1: Histological analysis of apoptosis, non-neuronal tissues, and additional neuronal subtypes. (**a**) Representative images of cross sections from the jejunum at 14 and 23 postconceptional weeks (PCW) with immunohistochemical (IHC) labeling against DAPI (white), pan-neuronal markers PGP9.5 (magenta) and HuC/D (green). White boxes indicate locations of higher magnitude insets. Brackets denote location of the submucosal plexus (SMP), circular muscle (CM), myenteric plexus (MP), and longitudinal muscle (LM). n = 2 14PCW and 3 23PCW. (**b**) Representative images of HuC/D labeling in wholemount preparation of the outer SMP in the small intestine at 15 and 20PCW. (**c-e**) Representative images of IHC labeling with apoptotic marker Caspase3 (magenta), HuC/D (green), and DAPI (white) in either (c) MP wholemount preparations at 15 and 20PCW or (d) cross sections at 14 and 23PCW. Yellow arrows indicate Caspase3+ cells. Scale bars as indicated. Percentage of HuC/D+ cells in the MP co-expressing Caspase3 in cross sections at 14 and 23PCW are shown in (e). Bars indicate means of data. (**f**) Representative images of smooth muscle marker SMA (magenta) and HuC/D (green) in cross sections of the MP from 14 and 23 PCW. Scale bars as indicated. (**g**) Thickness of SMA labeling of the CM and LM at 14 and 23PCW. Bars indicate means of data. (**h**) Representative images of glial and neural progenitor marker Sox10 (magenta) and HuC/D (green) in cross sections of the MP from 14 and 23 PCW. Scale bars as indicated. (**i**) Number of Sox10+ cells per area of MP in cross section at 14 and 23PCW. Bars indicate means of data. (**j-m**) IHC labeling in the jejunum MP with HuC/D (green) and sensory neuron marker NF-M (j, magenta) at 14 and 21PCW and interneuron marker SST (l, magenta) at 15 and 21PCW. White boxes indicate locations of higher magnitude insets. Proportion of total HuC/D+ neurons positive for NF-M (k) or SST (m) over developmental time. For NF-M, n = 1 PC 21-23PCW and DC 13-15PCW; n = 2 duo 21-23PCW, jej 21-23PCW, and PC 13-15PCW; n = 3 duo 18-20PCW, jej 18-20PCW, ileum 18-20PCW, ileum 21-23PCW, and DC 21-23PCW; n = 5 ileum 13-15PCW; n = 6 jej 13-15PCW; and n = 7 duo 13-15PCW. For SST, n = 1 ileum 13-15PCW; n = 2 duo 13-15PCW, duo 18-20PCW, duo 21-23PCW, jej 18-20PCW, jej 21-23PCW, ileum 18-20PCW, ileum 21-23PCW, PC 18-20PCW, PC 21-23PCW, and DC 21-23PCW; and n = 5 jej 13-15PCW. Trendlines interconnect means across age groups. Scale bars as indicated. Intestinal regions are noted with distinct colors as demonstrated in k. Source data are provided as a Source Data file.

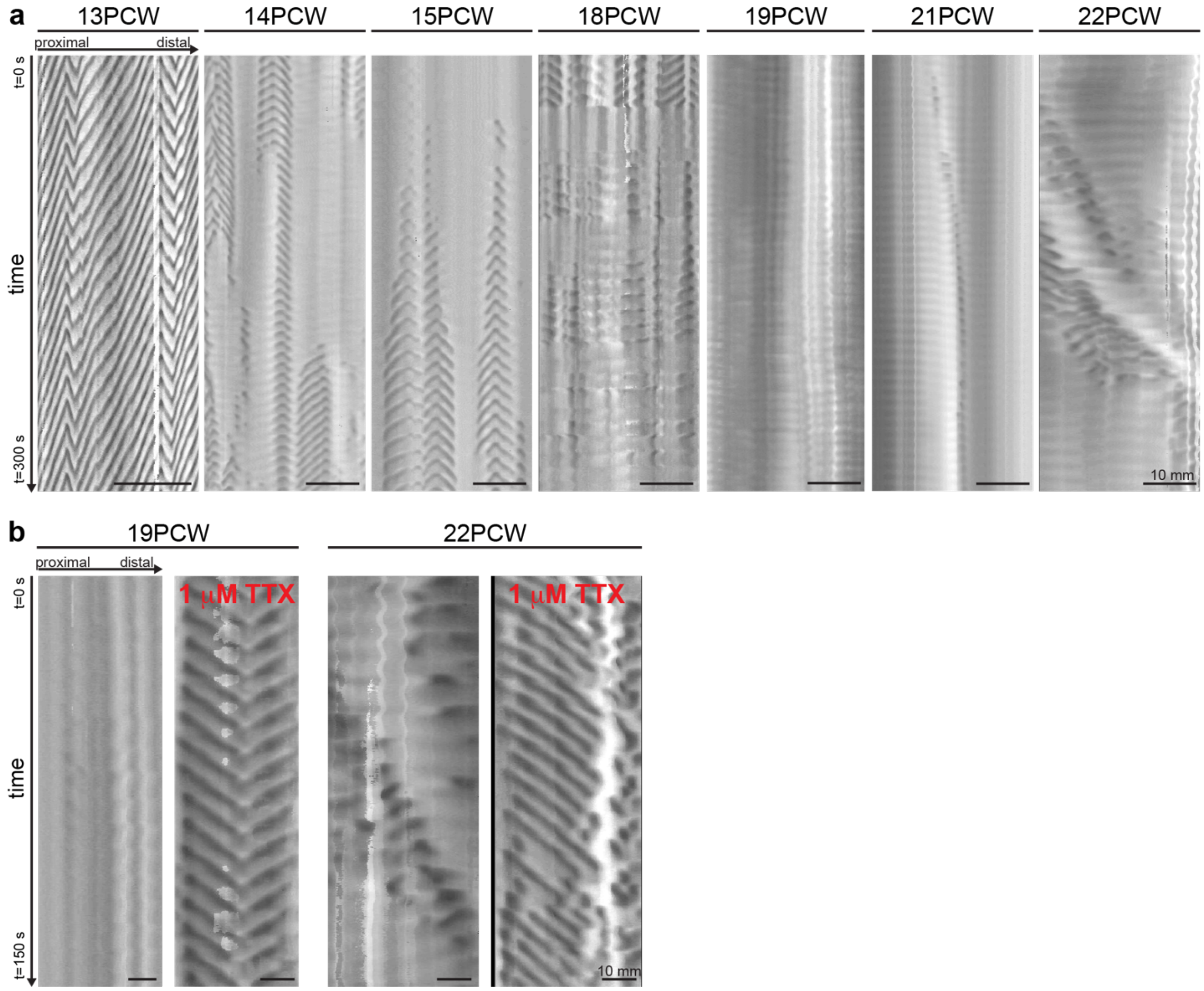

Supplementary Figure 2: *ex vivo* GI motility in the second trimester human fetal small intestine. (**a**) Spatiotemporal maps (STMs) with accompanying images of GI motility in the human fetal jejunum at ages spanning 13-22PCW. Dark gray: decreased intestinal diameter; light gray: increased. Scale bars as indicated. (**b**) STMs with representative images of GI motility in the human fetal duodenum at 19 and 22PCW at baseline (left panels) and with the addition of 1 μM tetrodotoxin (TTX, right panels). Dark gray: decreased intestinal diameter; light gray: increased. Scale bars as indicated.

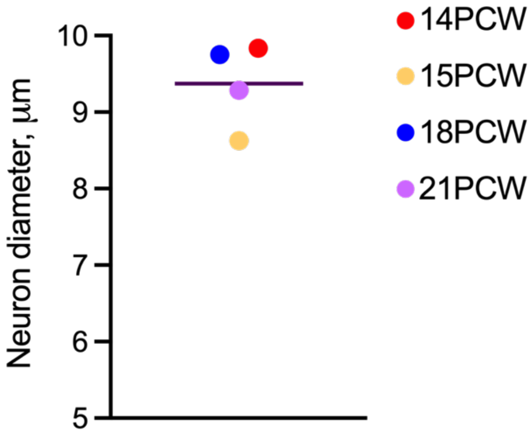

Supplementary Figure 3: Enteric neuron diameter. Average diameter of HuC/D+ enteric neurons across gestational age. n = 1 sample per age. Color indicates gestational age as indicated.

Supplementary Tables

Age (PCW)	Regions collected	Histology analyzed	Motility Analyzed
13.1	D, J	x	x
13.9	D,J	x	x
14	D, J, I, PC, DC	x	
14	D, J, I*, PC*, DC*	x	
14.7	D, J, I	x	
15	D, J, I	x	x
15	J, I	x	x
15.3	D, J	x	x
18	D, J, I, PC, DC	x	
18	D, J	x	
18	D, J	x	x
19	D, J, I	x	
19.3	D, J, I, PC, DC	x	
19.3	J, I, PC, DC	x	
19.4	D, J, PC, DC	x	x
20	D, J, I, PC	x	
21	D, J, I		x
21.1	D, J, I, PC, DC	x	
21.4	D, J, I, PC, DC	x	
21.9	D, J, I, PC, DC	x	x
22.5	D, J, I, PC, DC	x	
22.9	D, J, I, PC, DC	x	
23	D, J, I, PC, DC	x	

Supplementary Table 1: Samples collected. D, duodenum; J, jejunum; I, ileum PC, proximal colon; DC, distal colon. * indicates samples only analyzed as cross sections.

Antibody	Host	Dilution	Source	Catalogue No.	RRID

Calretinin	Goat	1:8000	Swant	CG1	AB_10000342
Caspase3	Rabbit	1:500	Abcam	AB2302	AB_302962
HuC/D	Mouse, biotinylated	1:500	ThermoFisher	A-21272	AB_2535822
Ki67	Rabbit	1:1000	Abcam	ab15580	AB_443209
Neurofilament-M	Rabbit	1:2000	Millipore	AB1987	AB_91201
nNOS	Rabbit	1:2000	Sigma-Aldrich	N7280	AB_260796
PGP9.5	Guinea pig	1:1000	Abcam	ab10410	AB_287150
Somatostatin	Rat	1:500	Millipore	MAB354	AB_2255365
Smooth muscle antigen	Goat	1:500	Abcam	ab21027	AB_1951138
Sox10	Goat	1:1000	R&D Systems	AF2864	AB_442208

Supplementary Table 2: Primary antibodies for immunohistochemistry

**Target**	**Fluorophore**	**Host**	**Dilution**	**Source**	**Catalogue No.**	**RRID**
Biotin	488	NA	1:1000	Invitrogen	S32354	NA
Guinea pig	Cy3	Donkey	1:800	Jackson Immuno Research	706-165-148	AB_2340460
Goat	Cy3	Donkey	1:800	Jackson Immuno Research	705-165-147	AB_2307351
Goat	Cy5	Donkey	1:800	Jackson Immuno Research	705-175-147	AB_2340415
Rabbit	Cy3	Donkey	1:800	Jackson Immuno Research	711-165-152	AB_2307443
Rabbit	Cy5	Donkey	1:800	Jackson Immuno Research	711-175-152	AB_2340607
Rat	Cy3	Donkey	1:800	Jackson Immuno Research	712-165-153	AB_2340667
Rat	Cy5	Donkey	1:800	Jackson Immuno Research	712-175-153	AB_2340672

Supplementary Table 3: Secondary antibodies for immunohistochemistry

Fig.1g z-score difference

**Ordinary one-way ANOVA summary**		
F		4.911
P value		0.0188
P value summary	*	
Significant diff. among means (P < 0.05	Yes	
R squared		0.6627

**ANOVA table**	**SS**	**DF**	**MS**	**F (DFn, DFd)**	**P value**
Treatment (between columns)	169.9	4	42.48	F (4, 10) = 4.911	P=0.0188
Residual (within columns)	86.49	10	8.649		
Total	256.4	14			

**Tukey's multiple comparisons test**	**Mean Diff.**	**95.00% CI of diff.**	**Summary**	**Adjusted P Value**
Duodenum vs. Jejunum	-1.326	-9.229 to 6.576	ns	0.9791
Duodenum vs. Ileum	-1.237	-9.140 to 6.666	ns	0.9838
Duodenum vs. PC	-7.805	-15.71 to 0.09789	ns	0.0533
Duodenum vs. DC	-7.504	-15.41 to 0.3982	ns	0.0647
Jejunum vs. Ileum	0.08951	-7.813 to 7.992	ns	>0.9999
Jejunum vs. PC	-6.478	-14.38 to 1.424	ns	0.1242
Jejunum vs. DC	-6.178	-14.08 to 1.725	ns	0.1495
Ileum vs. PC	-6.568	-14.47 to 1.335	ns	0.1174
Ileum vs. DC	-6.268	-14.17 to 1.635	ns	0.1415
PC vs. DC	0.3003	-7.602 to 8.203	ns	>0.9999

Fig.1i %Hu+CalR+

**Ordinary one-way ANOVA summary**		
F		15.59
P value		0.0006
P value summary	***	
Significant diff. among means (P < 0.05	Yes	
R squared		0.7392

**ANOVA table**	**SS**	**DF**	**MS**	**F (DFn, DFd)**	**P value**
Treatment (between columns)	79.14	2	39.57	F (2, 11) = 15.59	P=0.0006
Residual (within columns)	27.92	11	2.538		
Total	107.1	13			

**Tukey's multiple comparisons test**	**Mean Diff.**	**95.00% CI of diff.**	**Summary**	**Adjusted P Value**
13-15 vs. 18-20	-2.585	-5.307 to 0.1360	ns	0.0628
13-15 vs. 21-23	-5.967	-8.853 to -3.080	***	0.0004
18-20 vs. 21-23	-3.382	-6.268 to -0.4950	*	0.0226

Fig.1k %Hu+nNOS+

**Ordinary one-way ANOVA summary**		
F		1.769
P value		0.2122
P value summary	ns	
Significant diff. among means (P < 0.05	No	
R squared		0.2277

**ANOVA table**	**SS**	**DF**	**MS**	**F (DFn, DFd)**	**P value**
Treatment (between columns)	147.1	2	73.54	F (2, 12) = 1.769	P=0.2122
Residual (within columns)	499	12	41.58		
Total	646.1	14			

**Tukey's multiple comparisons test**	**Mean Diff.**	**95.00% CI of diff.**	**Summary**	**Adjusted P Value**
13-15 vs. 18-20	7.25	-3.631 to 18.13	ns	0.2183
13-15 vs. 21-23	5.794	-5.086 to 16.67	ns	0.3616
18-20 vs. 21-23	-1.455	-12.34 to 9.425	ns	0.9326

Fig.2d Ripple frequency

**Two-tailed paired t test summary**	**P value**	**Mean of Baseline**	**Mean of +Ttx**	**Difference**	**SE of difference**
13-15	0.552515	0.1283	0.1227	0.00564	0.008862
18,19	0.998849	0.09994	0.09998	-0.00003406	0.01885
21,22	0.170525	0.02837	0.08579	-0.05741	0.01576

Supplementary Fig.1k %Hu+NFM+

**Ordinary one-way ANOVA summary**					
F		0.09232			
P value		0.9126			
P value summary	ns				
Significant diff. among means (P < 0.05	No				
R squared		0.01813			

**ANOVA table**	**SS**	**DF**	**MS**	**F (DFn, DFd)**	**P value**
Treatment (between columns)	18.47	2	9.235	F (2, 10) = 0.0923	P=0.9126
Residual (within columns)	1000	10	100		
Total	1019	12			

**Tukey's multiple comparisons test**	**Mean Diff.**	**95.00% CI of diff.**	**Summary**	**Adjusted P Value**
13-15 vs. 18-20	3.105	-16.92 to 23.13	ns	0.9061
13-15 vs. 21-23	1.521	-15.82 to 18.86	ns	0.9687
18-20 vs. 21-23	-1.585	-21.61 to 18.44	ns	0.9745

Supplementary Fig.1m %Hu+SST+

**Ordinary one-way ANOVA summary**					
F		9.88			
P value		0.0054			
P value summary	**				
Significant diff. among means (P < 0.05	Yes				
R squared		0.6871			

**ANOVA table**	**SS**	**DF**	**MS**	**F (DFn, DFd)**	**P value**
Treatment (between columns)	3.163	2	1.582	F (2, 9) = 9.880	P=0.0054
Residual (within columns)	1.441	9	0.1601		
Total	4.604	11			

**Tukey's multiple comparisons test**	**Mean Diff.**	**95.00% CI of diff.**	**Summary**	**Adjusted P Value**	
13-15 vs. 18-20	0.1807	-0.6726 to 1.034	ns	0.8282	A-B
13-15 vs. 21-23	-0.929	-1.745 to -0.1131	*	0.0274	A-C
18-20 vs. 21-23	-1.11	-1.859 to -0.3602	**	0.0065	B-C

Supplementary Table 4: Pairwise statistical analyses.
