# Supplementary material for: Anatomical and functional maturation of the mid-gestation human enteric nervous system
Source: Nat Commun. 2023 May 9;14:2680. doi: 10.1038/s41467-023-38293-z (PMC10170115; doi:10.1038/s41467-023-38293-z)
Supplement: Supplementary file 2 — Reporting Summary [file 41467_2023_38293_MOESM2_ESM.pdf]

nature portfolio

Corresponding author(s): Julia A. Kaltschmidt

Last updated by author(s): Mar 13, 2023

Reporting Summary

Nature Portfolio wishes to improve the reproducibility of the work that we publish. This form provides structure for consistency and transparency in reporting. For further information on Nature Portfolio policies, see our Editorial Policies and the Editorial Policy Checklist.

Statistics

For all statistical analyses, confirm that the following items are present in the figure legend, table legend, main text, or Methods section.

n/a	Confirmed	
☐	☒	The exact sample size (*n*) for each experimental group/condition, given as a discrete number and unit of measurement
☐	☒	A statement on whether measurements were taken from distinct samples or whether the same sample was measured repeatedly
☐	☒	The statistical test(s) used AND whether they are one- or two-sided<br>*Only common tests should be described solely by name; describe more complex techniques in the Methods section.*
☒	☐	A description of all covariates tested
☐	☒	A description of any assumptions or corrections, such as tests of normality and adjustment for multiple comparisons
☐	☒	A full description of the statistical parameters including central tendency (e.g. means) or other basic estimates (e.g. regression coefficient) AND variation (e.g. standard deviation) or associated estimates of uncertainty (e.g. confidence intervals)
☐	☒	For null hypothesis testing, the test statistic (e.g. *F*, *t*, *r*) with confidence intervals, effect sizes, degrees of freedom and *P* value noted<br>*Give P values as exact values whenever suitable.*
☒	☐	For Bayesian analysis, information on the choice of priors and Markov chain Monte Carlo settings
☒	☐	For hierarchical and complex designs, identification of the appropriate level for tests and full reporting of outcomes
☒	☐	Estimates of effect sizes (e.g. Cohen's *d*, Pearson's *r*), indicating how they were calculated

Our web collection on statistics for biologists contains articles on many of the points above.

Software and code

Policy information about availability of computer code

Data collection	Confocal data was collected on a Leica SP8 running LASX software. Full details can be found in Methods.
Data analysis	Images were captured with Leica LASX. Image processing (e.g. cell counts) were performed in FIJI and Adobe Photoshop. Videos were captured with Imaging Source IC Capture (?) and analyzed with Volumetry G9a (Grant Hennig, University of Vermont). All statistics were performed in GraphPad Prism 9. Data analysis was performed in GraphPad Prism 9 and Jupyter Lab (Python) code previously described and available in Methods. Full details can be found in Methods.

For manuscripts utilizing custom algorithms or software that are central to the research but not yet described in published literature, software must be made available to editors and reviewers. We strongly encourage code deposition in a community repository (e.g. GitHub). See the Nature Portfolio guidelines for submitting code & software for further information.

Data

Policy information about availability of data

All manuscripts must include a data availability statement. This statement should provide the following information, where applicable:

- Accession codes, unique identifiers, or web links for publicly available datasets
- A description of any restrictions on data availability
- For clinical datasets or third party data, please ensure that the statement adheres to our policy

The data generated in this study are provided in Supplementary Information and Source Data files. Source data are provided with this paper. Video data generated in this study are provided in Supplementary Video files. Step-by-step protocols are available upon request. Correspondence and requests for all other material

should be addressed to L.B.D. or J.A.K.

Human research participants

Policy information about studies involving human research participants and Sex and Gender in Research.

Reporting on sex and gender	Sex data was not available at the time of the collection of human fetal tissue to protect patient privacy. Therefore, sex was not considered in this study.
Population characteristics	Samples consisted of intestinal tissue from pregnancies between 14 and 23 postconceptional weeks, based on a first trimester ultrasound and the date of the last menstrual period.
Recruitment	All women scheduled to have a pregnancy termination at Stanford were asked for consent to donate fetal tissue for research. All women who consented to donate tissue were eligible to participate in our study. All tissues collected were negative for trisomies, based on prenatal screening. Tissue was not collected from pregancies with prenatal screening positive for trisomies. All eligible, non-trisomy carrying samples were included in the study. To protect privacy, the age of the woman and the sex of the fetus were not made available to the research team.
Ethics oversight	Collection of fetal intestinal tissue was performed with approval from the Institutional Review Board at Stanford University. No monetary compensation was provided for consent to use fetal tissue as donation for research. Per the Stanford Institute Tissue Donation Policy, no study specific consent is need for the use of donated tissue that is not going to be cultured or used to create a cell line.

Note that full information on the approval of the study protocol must also be provided in the manuscript.

Field-specific reporting

Please select the one below that is the best fit for your research. If you are not sure, read the appropriate sections before making your selection.

[x] Life sciences [ ] Behavioural & social sciences [ ] Ecological, evolutionary & environmental sciences

For a reference copy of the document with all sections, see nature.com/documents/nr-reporting-summary-flat.pdf

Life sciences study design

All studies must disclose on these points even when the disclosure is negative.

Sample size	Samples were collected after elective pregnancy termination under a protocol approved through the Research Compliance Office at Stanford University. Given the sensitivity and rarity of this tissue, sample size was based on tissue availability.
Data exclusions	Samples were excluded from structural analysis if damage from the termination procedure and tissue collection disrupted our ability to identify individual enteric neurons. Regions of intestine found to have an incomplete myenteric plexus were excluded from motility analysis.
Replication	Each experiment included independent biological replicates if multiple samples of the same gestational age and intestinal region were available. The number of biological replicates for a given age, intestinal region, and experiment are indicated in associated figure legends. All attempts at replication were successful unless stated in "Data exclusions" above.
Randomization	Comparisons were made between regions of the intestines or between developmental time points, thus no randomization was necessary and groups were dictated by the nature of the sample.
Blinding	Given that our data were quantitative and not allocated into groups, blinding was not necessary for our analysis.

Reporting for specific materials, systems and methods

We require information from authors about some types of materials, experimental systems and methods used in many studies. Here, indicate whether each material, system or method listed is relevant to your study. If you are not sure if a list item applies to your research, read the appropriate section before selecting a response.

Materials & experimental systems

n/a	Involved in the study
[ ]	[x] Antibodies
[x]	[ ] Eukaryotic cell lines
[x]	[ ] Palaeontology and archaeology
[x]	[ ] Animals and other organisms
[x]	[ ] Clinical data
[x]	[ ] Dual use research of concern

Methods

n/a	Involved in the study
[x]	[ ] ChIP-seq
[x]	[ ] Flow cytometry
[x]	[ ] MRI-based neuroimaging

Antibodies

Antibodies used

Primary antibodies presented as: Target, Manufacturer and Catalogue Number, RRID
1. Calretinin Swant CG1 AB_10000342
2. Caspase3 Abcam AB2302 AB_302962
3. HuC/D ThermoFisher A-21272 AB_2535822
4. Ki67 Abcam ab15580 AB_443209
5. Neurofilament-M Millipore AB1987 AB_91201
6. nNOS Sigma-Aldrich N7280 AB_260796
7. PGP9.5 Abcam ab10410 AB_287150
8. Somatostatin Millipore MAB354 AB_2255365
9. Smooth Muscle Antigen Abcam AB_1951138
10. Sox10 R&D Systems AB_442208

Secondary antibodies include: Biotinylated 488 (Invitrogen S32354), Donkey anti-guinea pig Cy3 (Jackson Immuno Research 06-165-148), Donkey anti-goat Cy3 (Jackson Immuno Research 705-165-147), Donkey anti-goat Cy5 (Jackson Immuno Research 705-175-147), Donkey anti-rabbit Cy3 (Jackson Immuno Research 711-165-152), Donkey anti-rabbit Cy5 (Jackson Immuno Research 711-175-152), Donkey anti-rat Cy3 (Jackson Immuno Research 712-165-153), Donkey anti-rat Cy5 (Jackson Immuno Research 712-175-153).

Validation

1. Calretinin is knockout-validated in Schiffmann et al., 1999 (PMID: 10220453). Its use in the ENS has been extensively validated including by Misawa et al., 2010 (PMID 20677337) and Sanger et al., 2003 (PMID 12810847).
2. Caspase3 was validated to label apoptotic cells in human colonic tissue with immunofluorescence by manufacturer (Abcam).
3. HuC/D has been validated to label human neurons by the manufacturer (ThermoFisher) and has been shown to label human enteric neurons in studies including Walstab et al., 2014 (PMID24708203).
4. Ki67 is knockout-validated by manufacturer (Abcam) and has also been validated in human colonic tissue by manufacturer (Abcam).
5. Neurofilament-M was validated for use in human neurons by manufacturer (Millipore) and has been used to label subtypes of enteric neurons including in Rollo et al., 2015 (PMID: 26064478).
6. N-nitric oxide synthase has been shown to specifically label nitrergic neurons including in Kohlmeier et al., 2012 (PMID: 22956788) and has been used to label subtypes of enteric neurons in Hamnett et al., 2022 (PMID: 36070775).
7. PGP9.5 was validated in rat enteric neurons by the manufacturer (Abcam) and has been shown to label enteric neurons including in Chan et al., 2017 (PMID: 28671695).
8. Somatostatin was validated in human neurons by the manufacturer (Millipore) and has been utilized in the human duodenum in Miura et al., 2021 (PMID: 32839988).
9. Smooth muscle antigen was validated in the human duodenum by the manufacturer (Abcam).
10. Sox10 was validated in human cell lines by the manufacturer (R&D Systems) and has been extensively utilized in the enteric nervous system including in Rollo et al., 2015 (PMID: 28174705) and Agarwal et al., 2011 (PMID: 21610032).
