# Supplementary material for: Anatomical and functional maturation of the mid-gestation human enteric nervous system
Source: Nat Commun. 2023 May 9;14:2680. doi: 10.1038/s41467-023-38293-z (PMC10170115; doi:10.1038/s41467-023-38293-z)
Supplement: Supplementary file 4 — Description of Additional Supplementary Files [file 41467_2023_38293_MOESM4_ESM.pdf]

Description of Additional Supplementary Files

Supplementary Movie 1: *ex vivo* motility of the 14PCW jejunum. 10-minute video at 20x speed of a 30 mm segment of the 14 postconceptional week jejunum in the *ex vivo* gastrointestinal motility monitor. Tissue is oriented from proximal (left) to distal (right). Still images and accompanying spatiotemporal map for part of this video can be found in Figs.2a,b.

Supplementary Movie 2: *ex vivo* motility of the 18PCW jejunum. 10-minute video at 20x speed of a 30 mm segment of the 18 postconceptional week jejunum in the *ex vivo* gastrointestinal motility monitor. Tissue is oriented from proximal (left) to distal (right). Still image and accompanying spatiotemporal map for part of this video can be found in Fig.2a.

Supplementary Movie 3: *ex vivo* motility of the 21PCW jejunum. 10-minute video at 20x speed of a 30 mm segment of the 21 postconceptional week jejunum in the *ex vivo* gastrointestinal motility monitor. Tissue is oriented from proximal (left) to distal (right). Still image and accompanying spatiotemporal map for part of this video can be found in Fig.2a.

Supplementary Movie 4: *ex vivo* motility of the 22PCW jejunum. 10-minute video at 20x speed of a 30 mm segment of the 22 postconceptional week jejunum in the *ex vivo* gastrointestinal motility monitor. Tissue is oriented from proximal (left) to distal (right). Still image and accompanying spatiotemporal map for part of this video can be found in Fig.2a.

Supplementary Movie 5: *ex vivo* motility of the 14PCW duodenum at baseline. 10-minute video at 20x speed of a 25 mm segment of the 14 postconceptional week duodenum in the *ex vivo* gastrointestinal motility monitor prior to addition of tetrodotoxin. Tissue is oriented from proximal (left) to distal (right). Still image and accompanying spatiotemporal map for part of this video can be found in Fig.2c.

Supplementary Movie 6: *ex vivo* motility of the 14PCW duodenum with tetrodotoxin. 10-minute video at 20x speed of a 25 mm segment of the 14 postconceptional week duodenum in the *ex vivo* gastrointestinal motility monitor after the addition of 100 mM tetrodotoxin. Tissue is oriented from proximal (left) to distal (right). Still image and accompanying spatiotemporal map for part of this video can be found in Fig.2c.

Supplementary Movie 7: *ex vivo* motility of the 18PCW duodenum at baseline. 10-minute video at 20x speed of a 30 mm segment of the 18 postconceptional week duodenum in the *ex vivo* gastrointestinal motility monitor prior to addition of tetrodotoxin. Tissue is oriented from proximal (left) to distal (right). Still image and accompanying spatiotemporal map for part of this video can be found in Fig.2c.

Supplementary Movie 8: *ex vivo* motility of the 18PCW duodenum with tetrodotoxin. 10-minute video at 20x speed of a 30 mm segment of the 18 postconceptional week duodenum in

the *ex vivo* gastrointestinal motility monitor after the addition of 100 mM tetrodotoxin. Tissue is oriented from proximal (left) to distal (right). Still image and accompanying spatiotemporal map for part of this video can be found in Fig.2c.

Supplementary Movie 9: *ex vivo* motility of the 21PCW duodenum at baseline. 10-minute video at 20x speed of a 25 mm segment of the 21 postconceptional week duodenum in the *ex vivo* gastrointestinal motility monitor prior to addition of tetrodotoxin. Tissue is oriented from proximal (left) to distal (right). Still image and accompanying spatiotemporal map for part of this video can be found in Fig.2c.

Supplementary Movie 10: *ex vivo* motility of the 21PCW duodenum with tetrodotoxin. 10-minute video at 20x speed of a 25 mm segment of the 21 postconceptional week duodenum in the *ex vivo* gastrointestinal motility monitor after the addition of 100 mM tetrodotoxin. Tissue is oriented from proximal (left) to distal (right). Still image and accompanying spatiotemporal map for part of this video can be found in Fig.2c.
